# Reducing Stent-Induced Blooming Artifacts Using Virtual Monoenergetic Imaging Reconstructions in a Superficial Femoral Artery Phantom: An In-Vitro Study

**DOI:** 10.1007/s13239-026-00848-8

**Published:** 2026-07-07

**Authors:** Lisa Rutten, Lennart van de Velde, Cornelis H. Slump, Jasper Martens, Kartik Jain, Michel Versluis, Michel M. P. J. Reijnen

**Affiliations:** 1 Multi-Modality Medical Imaging Group, TechMed Centre, University of Twente, Hallenweg 5, 7522 NH Enschede, The Netherlands; 2 Department of Surgery, Rijnstate, Arnhem, The Netherlands; 3 Physics of Fluids group, TechMed Centre, University of Twente, Enschede, The Netherlands; 4 Department of Thermal and Fluid Engineering, University of Twente, Enschede, The Netherlands; 5 Robotics and Mechatronics Group, University of Twente, Enschede, The Netherlands; 6 Department of Radiology and Nuclear Medicine, Rijnstate, Arnhem, The Netherlands

**Keywords:** Spectral computed tomography, Virtual monoenergetic imaging, Blooming artifact, Stents, Peripheral arterial disease

## Abstract

**Purpose:**

Spectral computed tomography derived virtual monoenergetic imaging (VMI) reconstructions have been shown to reduce stent-induced blooming artifacts thus minimizing in-stent lumen underestimation. We have investigated the use of VMI reconstructions to reduce the blooming artifact for stent markers, single and double stent configurations.

**Methods:**

Two partially overlapping covered stents were deployed in a superficial femoral artery phantom. A conventional and twelve VMI reconstructions (40–100 keV and 120–200 keV with 10 and 20 keV increments, respectively) were created. Contrast-to-noise ratio, signal-to-noise ratio, attenuation difference between native and in-stent vessel lumen and full width at half maximum (FWHM) of the stent peaks were evaluated. The in-stent lumen diameter was measured, and the stent evaluated using a 5-point scale by three interventional radiologists.

**Results:**

The 80–100 keV VMI reconstructions for the single stent configuration and the 140 keV VMI reconstruction for the other configurations resulted in the smallest lumen underestimation and best quality score on blooming. The underestimation was reduced from 21.5–29.1% for the conventional reconstruction to 14.1–18.6% for the optimal VMI reconstructions. No significant differences were observed in FWHM between the conventional reconstruction and the 50–200 keV VMI reconstructions.

**Conclusions:**

VMI was found to minimize stent-induced blooming artifacts and improve in-stent lumen assessment. More stent material requires higher-energy VMI reconstructions to reduce blooming. The absence of significant differences in quantitative parameters suggests that the number of pixels affected by the blooming artifact is not reduced using VMI reconstructions.

**Supplementary Information:**

The online version contains supplementary material available at https://doi.org/10.1007/s13239-026-00848-8.

## Introduction

Despite advances in endovascular therapy, the occurrence of in-stent restenosis remains to be an important limitation in the femoropopliteal artery with an incidence of 15–40% within one year after treatment [1]. Computational fluid dynamics (CFD) simulations derived flow parameters can be used to predict in-stent restenosis and can be used as a tool for improving patient treatment or device design that could in the end prevent in-stent restenosis [2]. To obtain CFD-based in-stent restenosis predictions, accurate lumen segmentations, often based on computed tomography angiography (CTA), are required. Quantitative evaluation of CTA scans with stents in place, however, is limited by stent-induced blooming artifacts that cause erroneous in-stent lumen narrowing [3], resulting in inaccurate lumen segmentations and thus impacting the reliability of CFD simulations [4].

The effect of blooming artifacts on the in-stent lumen in frequently used superficial femoral artery stents was investigated by Kaempf et al. (2012) [5]. The visible lumen diameter was underestimated by 16.7 and 22.4% for the Viabahn (W.L. Gore and associates) and Everflex stent (Medtronic), respectively [5]. Around proximal and distal radiopaque stent markers, that are used to improve stent visibility during stent deployment, the blooming artifact was even more pronounced. For an Astron stent (Biotronik), the visible lumen underestimation was 31.8% at the proximal and distal radiopaque stent markers compared to 18.8% for the other stented areas [5]. Stent-in-stent placement, often required in the longer femoropopliteal lesions, also caused more blooming due to the double stent layer, however, this was not quantified [6].

Blooming artifacts can be reduced using spectral computed tomography (CT)-derived virtual monoenergetic imaging (VMI) reconstructions [7–9]. In high kiloelectronvolt (keV) VMI reconstructions (> 100 keV), the absorption of materials with high-atomic-number elements, such as nitinol stent struts, is minimal, resulting in less blooming. However, this happens at the cost of image contrast. Studies investigating the effect of VMI reconstructions on the blooming artifact report conflicting results based on different parameters, including the attenuation difference between the in-stent lumen and native vessel lumen [10], full width at half maximum (FWHM) of the stent struts [11], quantitative and qualitative image quality parameters [10, 12] and in-stent lumen diameter measurements by radiologists [11, 12]. Two studies [10, 12] identified the 80–100 keV energy range as the optimal balance between artifact reduction and image quality, while another study [11] did not find an effect of VMI reconstructions on quantitative artifact reduction. Furthermore, previous studies only focused on a single stent configuration, while both radiopaque stent markers and a stent-in-stent configuration are commonly used in the femoropopliteal tract. Therefore, in this study the VMI reconstruction that best reduces blooming while preserving lumen assessment in a single stent, double stent and radiopaque stent marker configuration is investigated in a superficial femoral artery phantom, using quantitative and qualitative parameters.

## Methods

### Experimental Setup

**Fig. 1:**
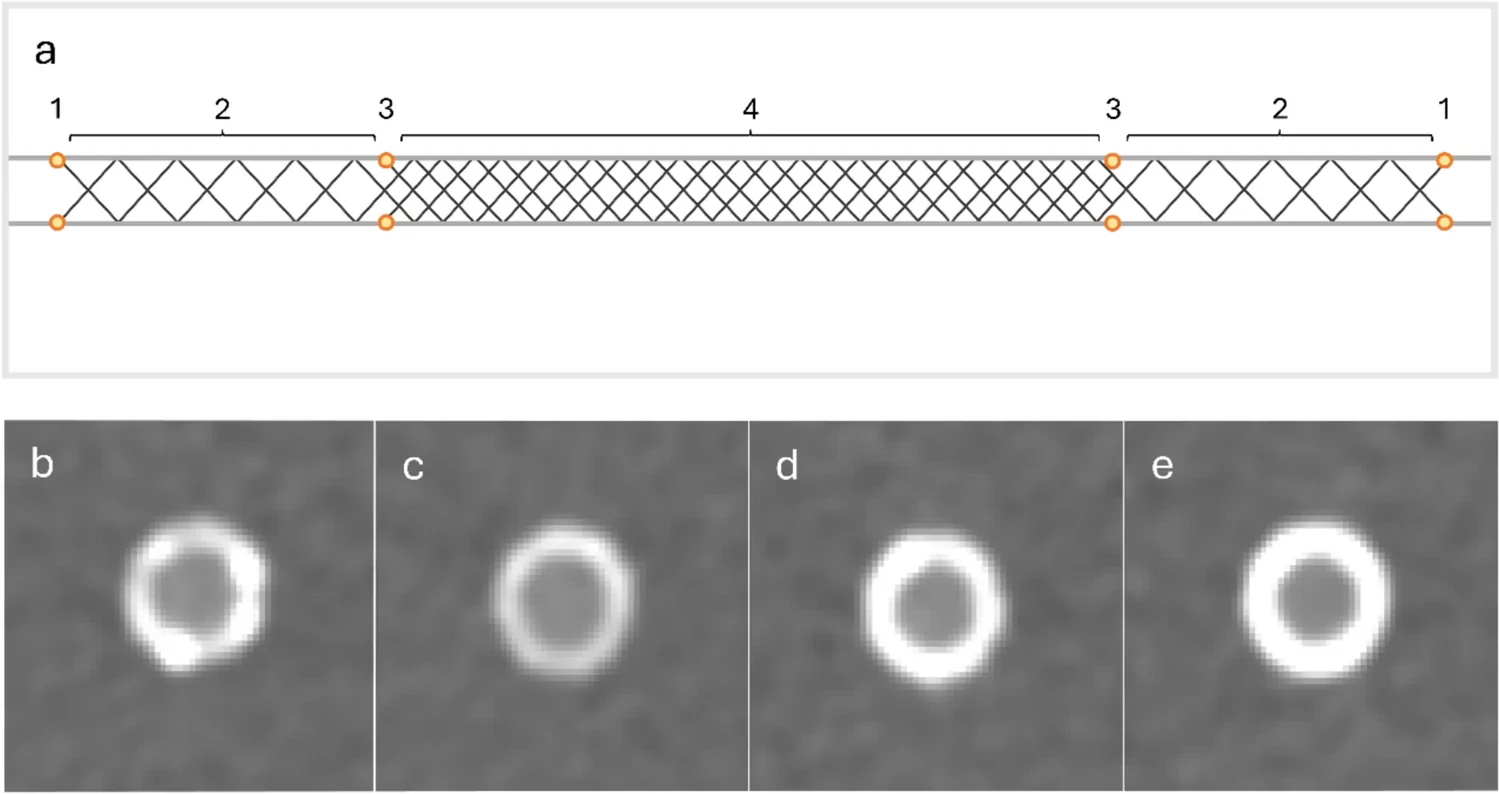
Experimental setup. **a** Schematic overview of the tube with two partly overlapping stents with radiopaque stent markers (yellow dots) and the resulting analysis locations: (1) single stent + marker configuration, (2) single stent configuration, (3) double stent + marker configuration, (4) double stent configuration. **b**–**e** iDose image reconstructions with default window settings (center: 300 HU, width: 1500 HU) for analysis locations 1–4, respectively

Two self-expanding covered stents (Viabahn, W.L. Gore and associates) with a nominal diameter of 6.0 mm, a length of 150 mm and strut thickness of 0.150 mm were analyzed in an in-vitro setup. An optically transparent polydimethylsiloxane (PDMS, Sylgard 184, Dow Corning) phantom with dimensions 220 × 85 × 65 mm^3^ and two cylindrical channels with a diameter of 5.0 mm, was created to mimic two superficial femoral arteries and the surrounding tissue. The self-expanding covered stents were deployed by eye by an experienced vascular surgeon (MR) in one of the channels with 100 mm overlap, resulting in one channel without stents and one channel with two partially overlapping stents (stent-in-stent configuration). This resulted in four analysis locations with stent: single stent configuration, single stent + marker configuration, double stent configuration and double stent + marker configuration, see Fig. 1a. The channels were filled with Xenetix 300 (Guerbet) in a ratio of 1:30 with water to obtain CT values of 250–300 HU at 120 kilovoltage peak (kVp). The phantom was placed parallel to the longitudinal axis of the CT scanner to mimic the orientation of a subject’s superficial femoral artery inside a CTA scan.

### Acquisition Parameters

**Fig. 2:**
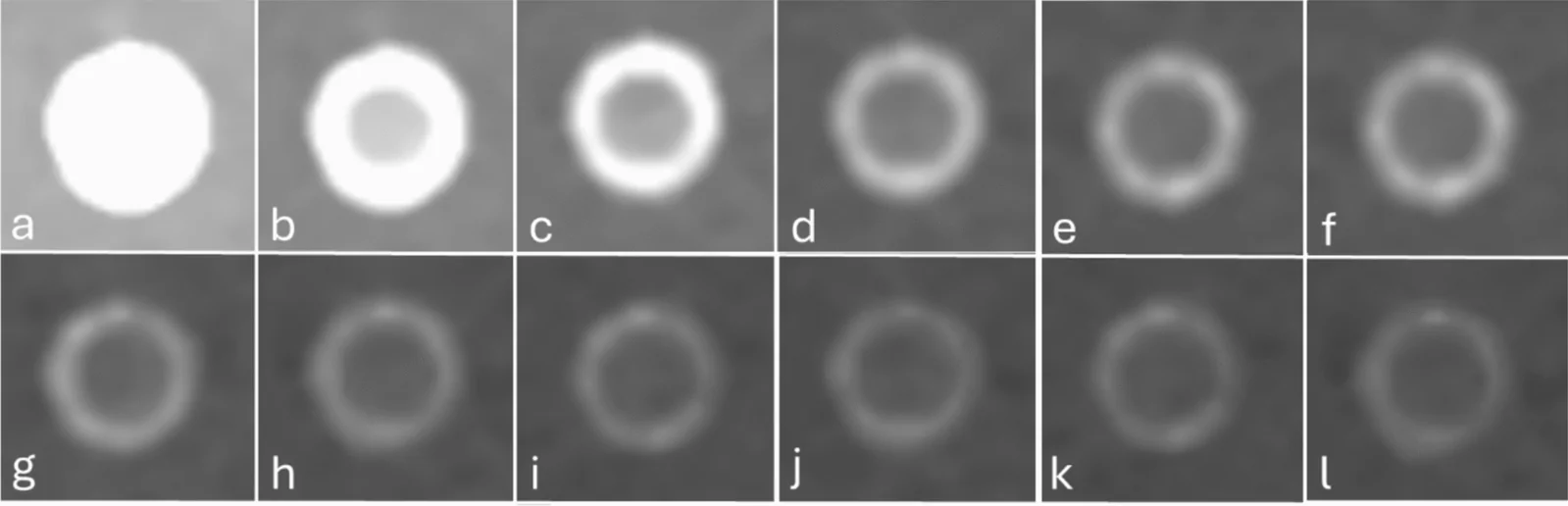
Virtual monoenergetic imaging (VMI) reconstructions for the single stent configuration. **a**–**g** 40–100 keV VMI reconstructions with increments of 10 keV, **h**–**l** 120–200 keV VMI reconstructions with increments of 20 keV. All reconstructions are presented with default window settings (center: 300 HU, width: 1500 HU)

All CTA data was acquired on a dual-layer detector-based spectral CT scanner (IQon, Philips Healthcare). The following scanning parameters were used: collimation, 64 × 0.625 mm; rotation time, 0.75 s; tube current-time product, 15 mAs; tube voltage, 120 kVp. All reconstructions were created using an iterative reconstruction algorithm (iDose level3, Philips Healthcare) with a slice thickness of 0.9 mm and an increment of 0.45 mm. The pixel matrix contained 512 × 512 pixels with a pixel size of 0.24 × 0.24 mm^2^. A conventional reconstruction (iDose, see Fig. 1b–e), and twelve VMI reconstructions (40–100 keV with increments of 10 keV, 120–200 keV with increments of 20 keV, see Fig. 2), were reconstructed (IntelliSpace Portal, Philips Healthcare). For initial image analysis fixed window settings were used (width 1500 HU, center 300 HU).

The phantom was scanned a second time with a tube voltage of 120 and 140 kVp to analyze the effect of the tube voltage on VMI reconstructions.

### Data Analysis

**Fig. 3:**
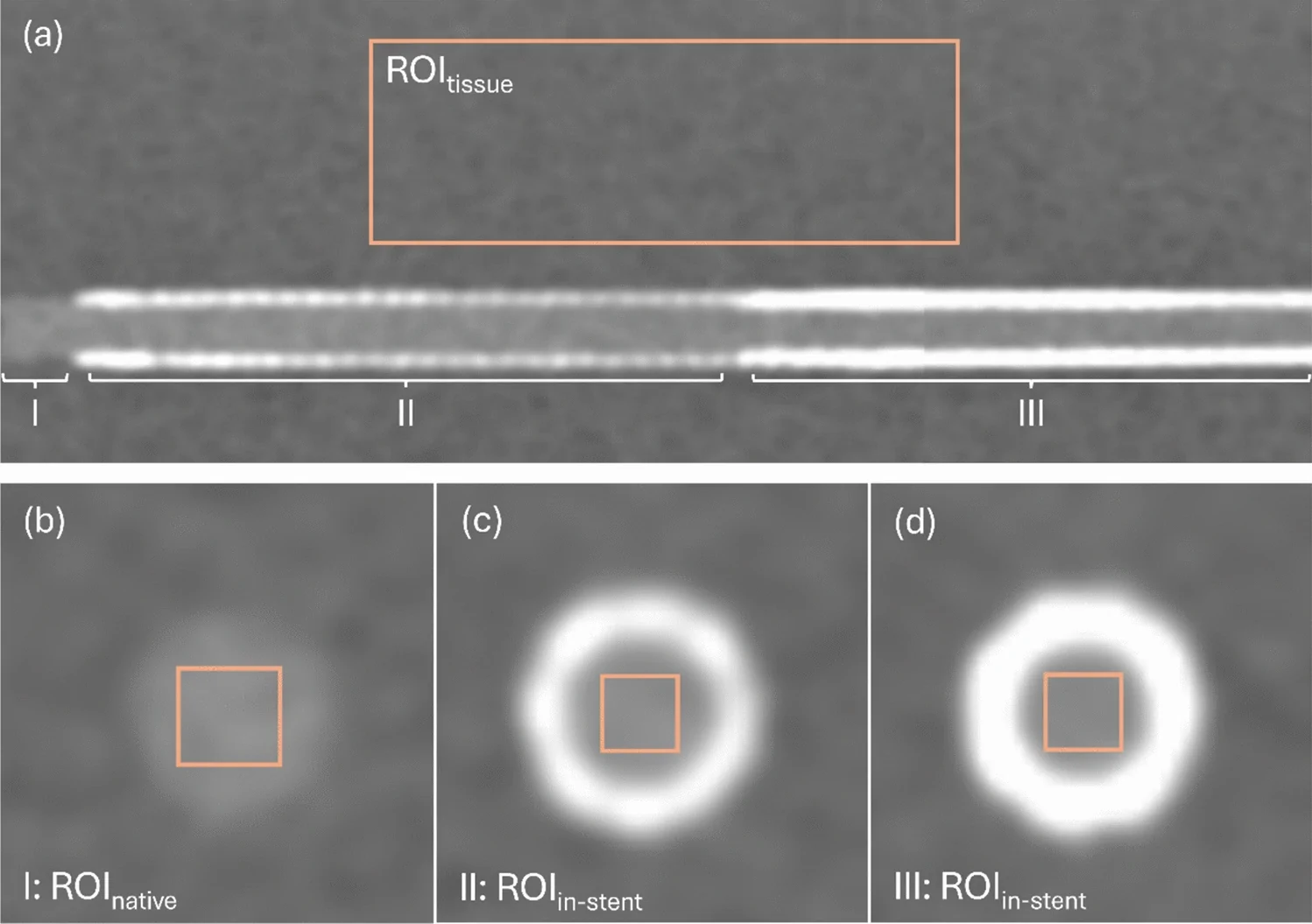
Overview of the regions of interest (ROI). **a** ROI in the tissue (ROI_tissue_) and different positions in the vessel lumen: (I) native vessel lumen proximal to the stent, (II) single stented lumen, (III) double stented lumen. **b** ROI in the native vessel proximal to the stent (ROI_native_), **c** and **d** ROI in the single and double stented lumen (ROI_in-stent_)

Data analysis was performed in MATLAB R2021b (The MathWorks). For the stent-marker configurations six slices and for no stent-marker configurations ten slices, were analyzed (Fig. S1). Three types of regions of interest (ROI) were defined: an ROI in the tissue (ROI_tissue_), an ROI in the native vessel lumen, either proximal or distal to the stent (ROI_native_) and an ROI in the in-stent vessel lumen (ROI_in-stent_), see Fig. 3. All lumen ROIs were chosen as large as possible, carefully excluding the stent or (blooming) artifacts by positioning the ROI for each slice separately, resulting in 25 voxels per ROI for the double stent + marker configuration and 36 voxels for the other stent configurations. The data was analyzed for the ROI in all slices collectively per analysis location. The image quality was quantified using image noise, signal-to-noise ratio (SNR) and contrast-to-noise ratio (CNR). Image noise was defined as the standard deviation (SD) in the tissue (SD_tissue_). The SNR and CNR were calculated as follows:

$${\mathrm{SNR}} = \frac{{{\mathrm{HU}}_{{{\mathrm{in}} - {\mathrm{stent}}}} }}{{{\mathrm{SD}}_{{{\mathrm{in}} - {\mathrm{stent}}}} }},$$

$${\mathrm{CNR}} = \frac{{\left( {{\mathrm{HU}}_{{{\mathrm{in}} - {\mathrm{stent}}}} - {\mathrm{HU}}_{{{\mathrm{tissue}}}} } \right)}}{{{\mathrm{SD}}_{{{\mathrm{tissue}}}} }},$$

where HU_x_ is the mean and SD_x_ the standard deviation in the corresponding ROI.

The attenuation difference, defined as HU_in-stent_–HU_native_, was calculated for all analysis locations to quantify the amount of blooming per reconstruction, as was used before [10, 13]. Furthermore, for every analysis location a straight line was semi-automatically drawn across the diameter of the vessel. This line was drawn in six slices for the stent-marker configurations and in ten slices for the no stent-marker configurations. From the resulting intensity profiles, the full width at half maximum (FWHM) of both peaks was determined (Fig. S2) and then averaged. The averaged FWHM per analysis location and energy level was used as an alternative method to quantify the blooming effect for each reconstruction.

Based on the previous data analysis, eight reconstructions (iDose, 40 keV, 60–100 keV with increments of 10 and 140 keV) per analysis location were assessed by three interventional radiologists experienced with the interpretation of cardiovascular CTA scans. The in-stent lumen diameter was measured, and the image was scored on (i) clarity of stent-lumen interface, (ii) presence of blooming artifact and (iii) overall image quality using a 5-point Likert scale [14] (Table 1). The reconstructions were presented in a random order with fixed window settings. After initial scoring, the readers could choose to change the window settings. During scoring, the readers were blinded to the reconstruction method and each other’s results.

**Table 1:** 5-point Likert scale for stent evaluation on stent-lumen interface, blooming artifact and image quality

Score	Stent-lumen interface	Blooming artifact	Image quality
5	Very clear visualization of stent structure and vessel lumen.	No blooming artifact present	Excellent image quality
4	Slightly blurred stent-lumen interface	Minimal blooming artifacts present	Good image quality
3	Somewhat blurred stent-lumen interface	Moderate blooming artifacts present	Moderate image quality
2	Blurred stent-lumen interface	Marked blooming artifacts present	Poor image quality
1	Stent and lumen cannot be distinguished	Severe blooming artifacts present	Very poor image quality, in-stent lumen cannot be assessed

### Statistical Analysis

IBM SPSS statistics 28.0 (IBM corporation) was used for statistical analysis. Data is presented as median (interquartile range), unless specified otherwise. The interobserver agreement of the three readers was calculated for the diameter measurements using the intraclass correlation coefficient based on absolute-agreement, two-way mixed-effects models and for the qualitative scoring using Fleiss’ kappa (for three or more raters). Quantitative and qualitative differences between reconstructions were tested for statistical significance using Friedman’s ANOVA with post-hoc Bonferroni correction as the non-parametric alternative to the repeated-measured ANOVA, given the small sample sizes. A p-value < 0.05 was considered statistically significant.

## Results

### Imaging Parameters

**Fig. 4:**
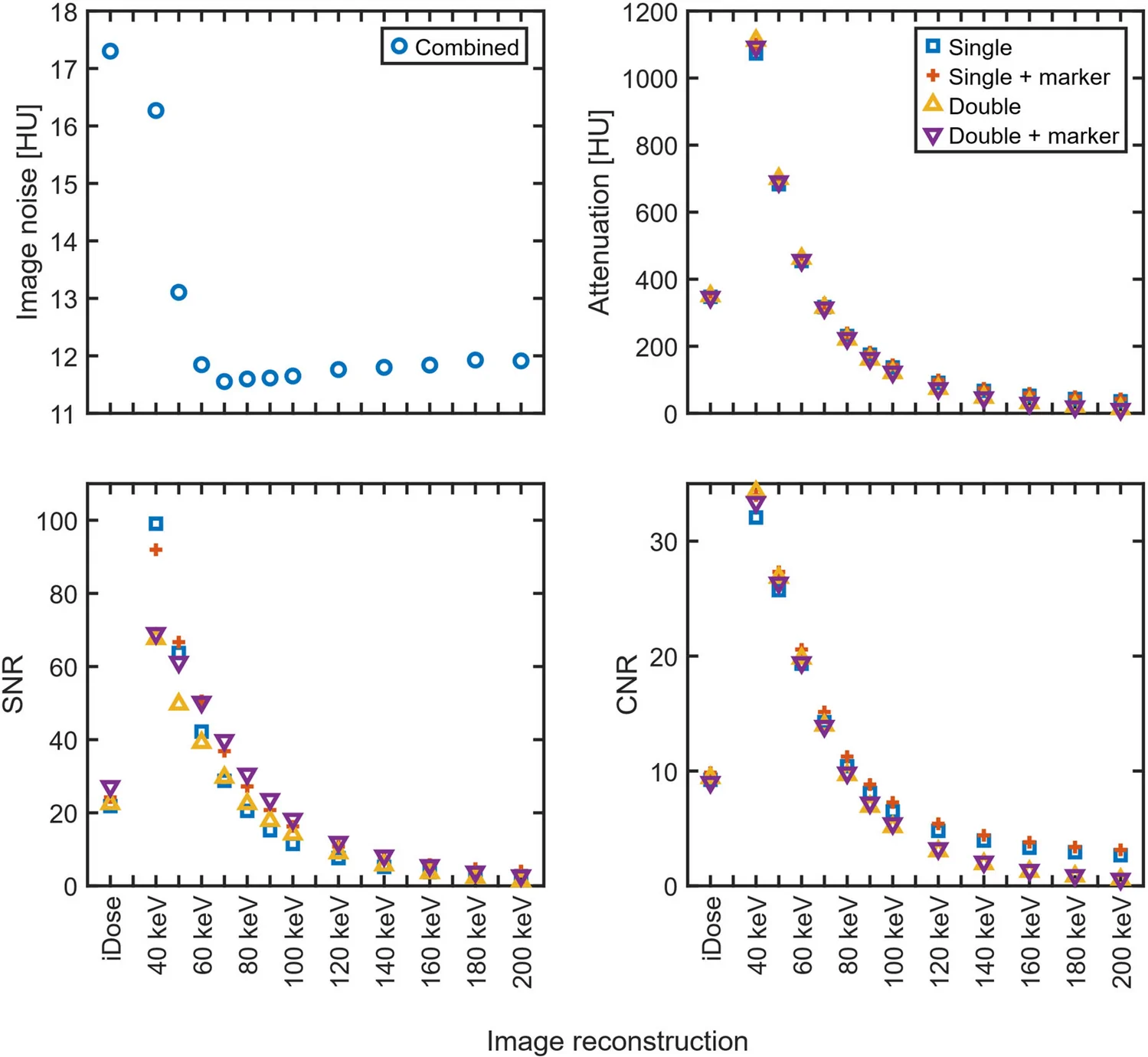
The median image noise (**a**), in-stent lumen attenuation (**b**), signal-to-noise ratio (SNR, **c**) and contrast-to-noise ratio (CNR, **d**) for conventional and virtual monoenergetic image (VMI) reconstructions. For the image noise, data from all analyzed slices was combined. The in-stent lumen attenuation, SNR and CNR are provided for the different stent configurations: single stent, single stent + marker, double stent, and double stent + marker

Figure 4 displays the evaluation of the imaging parameters for the different energy levels and conventional reconstruction. The image noise (Fig. 4a) in the iDose and 40 keV VMI reconstruction was significantly higher than in all other reconstructions (p < 0.05). The image noise decreased with increasing energy level until the 70 keV VMI reconstruction after which a slight increase in image noise with increasing energy level was observed. The in-stent lumen attenuation (Fig. 4b), SNR (Fig. 4c) and CNR (Fig. 4d) decreased with an increasing energy level for all stent configurations. The iDose reconstruction was scored between the 60–70 keV VMI reconstructions on in-stent lumen attenuation and between the 70–90 keV VMI reconstructions on the SNR and CNR.

### Attenuation Difference

**Fig. 5:**
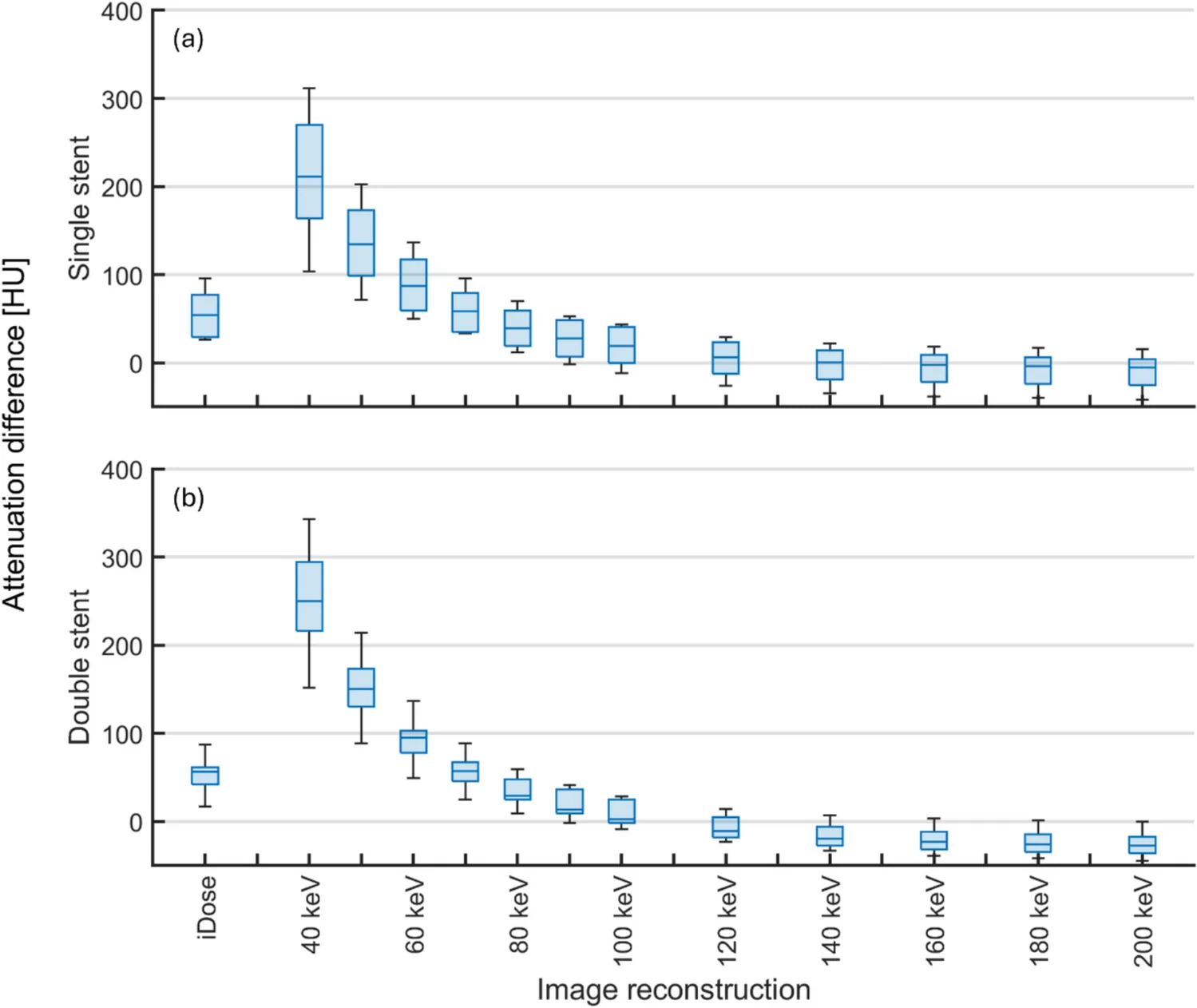
The attenuation difference between in-stent lumen and native vessel lumen in the single stent (**a**) and double stent (**b**) configurations for conventional and virtual monoenergetic image (VMI) reconstructions. The median attenuation difference is negative in the single stent configuration for the 160–200 keV VMI reconstructions and in the double stent configuration for the 120–200 keV VMI reconstructions

The attenuation difference between the in-stent lumen and native vessel lumen for the single stent and double stent configurations are depicted in Fig. 5 and for the stent + marker configurations in supplementary Fig. S3. For all stent configurations the attenuation difference decreased with increasing energy level. The attenuation difference was negative in the 160–200 keV energy range for the single stent configuration (median: − 2.1 to − 5.0 HU) and in the 120–200 keV energy range for the double stent (median: − 11.1 to − 27.5 HU) and double stent + marker configuration (median: − 10.8 to − 28.5 HU). The attenuation difference in the iDose reconstruction was scored between the 70–80 keV VMI reconstructions for all stent configurations. Supplementary Fig. S4 displays the attenuation difference for the single and double stent configurations at 120 and 140 kVp, showing that the attenuation difference is smaller for the scans acquired at 140 kVp compared to 120 kVp.

### Blooming Effect (FWHM)

**Fig. 6:**
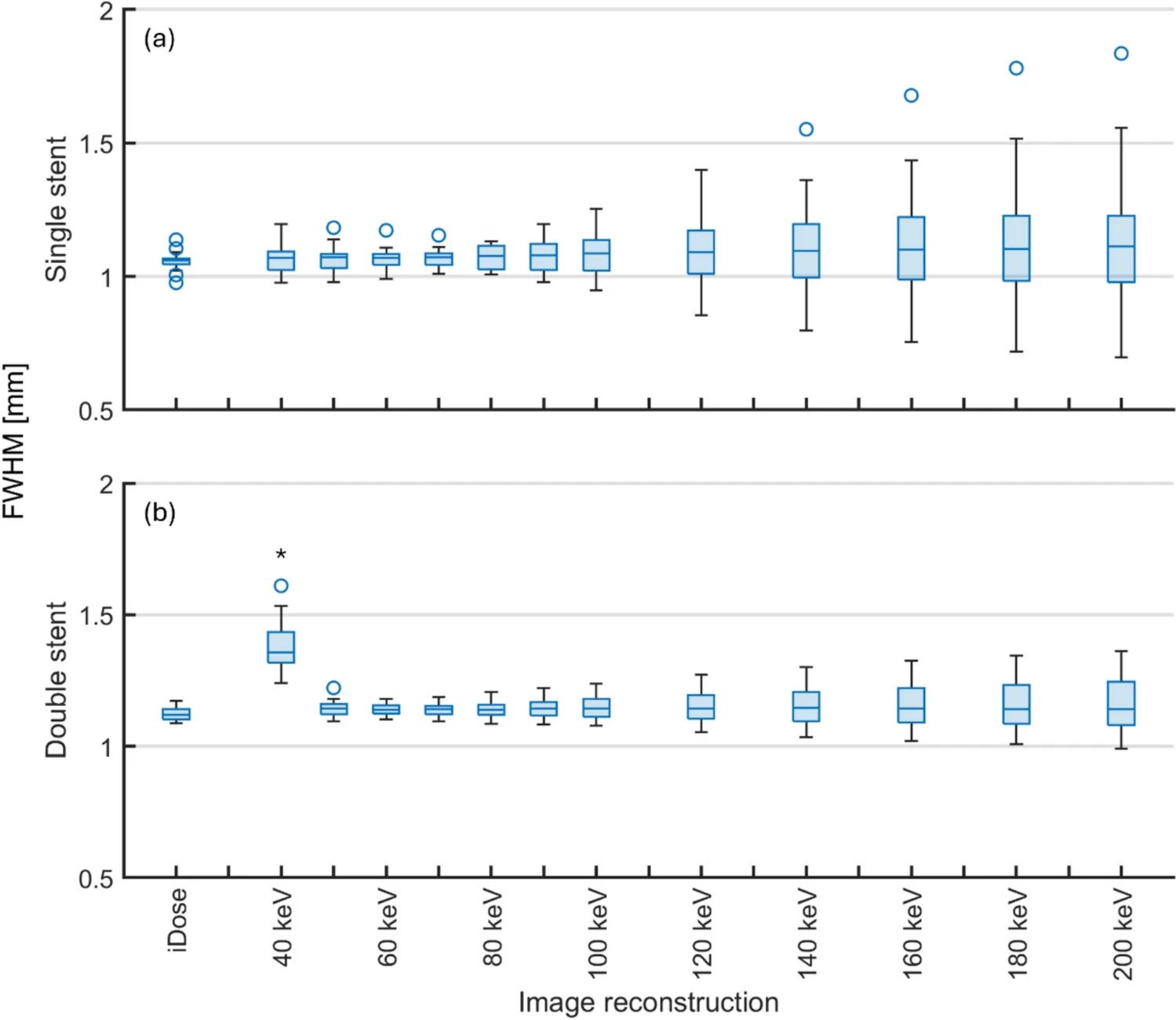
The full width at half maximum (FWHM) in the single (**a**) and double stent (**b**) configurations for conventional and virtual monoenergetic image (VMI) reconstructions. The circles are outliers defined as more than 1.5 × interquartile range away from the top or bottom of the box. *significantly larger than iDose, VMI 60–90 keV and 120 keV (p < 0.05)

Figure 6 shows the FWHM in the single and double stent configurations and supplementary Fig. S5 presents the data for the stent + marker configurations for conventional and VMI reconstructions. The FWHM in the 40 keV VMI reconstruction was significantly larger than in the iDose, 60–90 and 120 keV VMI reconstructions for the double stent configuration and significantly larger than in the 180–200 keV VMI reconstructions for the double stent + marker configuration. For the other reconstructions and stent configurations, the interquartile range was equal to or smaller than the pixel size (0.24 mm), except for the 40 keV VMI reconstruction in the single stent + marker configuration, and the differences in FWHM were not statistically significant.

### In-Stent Lumen Diameter

Table 2 summarizes the results of the in-stent lumen diameter measurements by independent readers for conventional and VMI reconstructions. The interobserver agreement for the measured in-stent lumen diameter was good (intraclass correlation coefficient: 0.75). The readers adjusted the window settings for the 40 keV VMI reconstruction (eight times) as otherwise the diameter could not be measured. The measured in-stent lumen diameter was closest to the true in-stent lumen diameter at 80 keV for the single stent configuration (82.2% of true lumen diameter) and at 140 keV for the other three stent configurations (81.4–85.9% of true lumen diameter). The measured diameter in the iDose reconstruction was between 70.9 and 78.5% of the true lumen diameter. No statistically significant differences in measured lumen diameter were found between the reconstructions for all analysis locations.

**Table 2:** In-stent lumen diameter (in mm (top row) and in percentage of true lumen diameter (bottom row)) measured by three independent readers for conventional and virtual monoenergetic image reconstructions

	iDose	40 keV	60 keV	70 keV	80 keV	90 keV	100 keV	140 keV
Single, mm (%)	3.61 (78.5)	3.33 (72.4)	3.52 (76.5)	3.48 (75.7)	3.78 (82.2)	3.72 (80.9)	3.74 (81.3)	3.70 (80.4)
Single + marker, mm (%)	3.32 (72.2)	2.96 (64.3)	3.21 (69.8)	3.61 (78.5)	3.54 (77.0)	3.78 (82.2)	3.70 (80.4)	3.84 (83.5)
Double, mm (%)	3.39 (77.0)	3.04 (69.1)	3.16 (71.8)	3.52 (80.0)	3.65 (83.0)	3.56 (80.9)	3.75 (85.2)	3.78 (85.9)
Double + marker, mm (%)	3.12 (70.9)	3.00 (68.2)	2.86 (65.0)	3.26 (74.1)	3.57 (81.1)	3.47 (78.9)	3.57 (81.1)	3.58 (81.4)

The measured in-stent lumen diameter closest to the true in-stent lumen diameter (4.6 mm for the single stent and single stent + marker configurations; 4.4 mm for the double stent and double stent + marker configurations) is underlined.

### Image Quality Score

The combined Likert scores on stent evaluation are summarized in Table 3 and the scores for stent-lumen interface, blooming artifact and image quality are presented in supplementary Table S1–3 for the conventional and VMI reconstructions. The combined inter-observer agreement for assessing the stent-lumen interface, blooming artifact and image quality was fair to moderate (Fleiss Kappa: 0.42 [0.34–0.49]). The combined score on stent evaluation was highest in the 90–100 keV energy range for all stent configurations, except for the double stent configuration where the highest score was found at 140 keV. For the stent-lumen interface and image quality the scores were generally highest for the 90-100 keV. For the blooming artifact, maximum Likert scores were mostly given for the 140 keV VMI reconstruction. The iDose reconstruction scored between the 60–80 keV VMI reconstructions for all stent configurations. The lowest Likert scores were found in the lower energy range (40–60 keV).

**Table 3:** Mean image quality scores on stent evaluation (Likert scale 1–5) based on three independent readers for conventional and virtual monoenergetic image (VMI) reconstructions

	iDose	40 keV	60 keV	70 keV	80 keV	90 keV	100 keV	140 keV
Single	2.78	1.00	2.00	2.33	3.67^*,^^	3.78^*,^^	4.00^*,^,~^	3.33^*,^^
Single + marker	2.33	1.00	2.11	2.78	3.33^*^	4.00^*,^,†^	3.67^*,^^	3.78^*,^^
Double	2.00	1.00	1.78	2.11	3.11^*^	2.56	3.67^*,^^	4.00^*,^,~,†^
Double + marker	2.00	1.00	1.78	2.11	3.00^*^	3.44^*,^,†^	3.22^*,^^	3.33^*,^^

Significant differences (p < 0.05) are indicated as: *significantly higher than VMI 40 keV, ^significantly higher than VMI 60 keV, ~significantly higher than VMI 70 keV, †significantly higher than iDose. The highest agreement per stent configuration is underlined

## Discussion

The optimal keV VMI reconstruction to reduce blooming in a stented superficial femoral artery phantom was dependent of the stent configuration. Visual inspection by interventional radiologists revealed the largest in-stent lumen diameter measurements and Likert score on blooming in the 140 keV VMI reconstruction for the stent + marker configurations and the double stent configuration, compared to the 80–100 keV VMI reconstruction for the single stent configuration. These results suggest that in case of more stent material, higher energy levels are required to qualitatively minimize the blooming artifact. Using these VMI reconstructions reduced the in-stent lumen diameter underestimation from 21.5 to 29.1% for the conventional reconstruction to 14.1–18.6% for the optimal VMI reconstructions, enabling more accurate in-stent restenosis assessment. The FWHM, however, showed no significant differences between the conventional and 50–200 keV VMI reconstructions, suggesting that the number of pixels affected by blooming, as well as the image intensity gradient, was not reduced.

VMI reconstructions in the low energy range (40–60 keV) were characterized by high SNR and CNR values as they benefit from the proximity to the iodine K-edge (33 keV) [15] and low image noise [16], as was also observed before [12, 13]. At the same time, the photoelectric effect [17] enhances blooming in the lower energy VMI reconstructions. This was confirmed by the high attenuation difference, similar to results of previous studies [10, 13], and by the poor Likert scores on stent evaluation in the lower energy VMI reconstructions. In the high energy range (120–200 keV), on the other hand, beam hardening artifacts are minimized, resulting in less blooming [7]. This was shown by the reduced attenuation difference and improved Likert score on blooming, although at the cost of reduced CNR values, that could severely hamper in-stent restenosis assessment [18]. Combining the above, the optimal VMI reconstruction for the single stent configuration was in the 80–100 keV range based on in-stent lumen diameter and Likert scores, which is in line with previous studies [10, 12]. More stent material required more blooming artifact reduction, shifting the optimal range to 100–140 keV for the double stent configuration. For the stent + marker configurations, optimal scores were obtained at 80 and 140 keV, depending on whether image quality or blooming artifact was scored.

No significant differences were observed in FWHM between the conventional reconstruction and the 50–200 keV VMI reconstructions for all stent configurations. Halpern et al. (2009) [11] and Park et al. (2024) [19] also found no significant differences in FWHM and full width at one-third of the maximum between different VMI reconstructions. These results suggest that the number of pixels affected by the blooming artifact is not reduced by VMI reconstructions, thereby limiting their applicability for improving segmentations for CFD simulations. Blooming artifacts are the results of both beam hardening artifacts and partial volume averaging [6]. The first can be reduced by reconstructing high-energy VMI reconstructions, while the latter is not affected by VMI reconstructions. Due to the limited pixel width of 0.24 mm, compared to a strut thickness of 0.15 mm, underestimation of the in-stent lumen diameter will remain in the VMI reconstructions. To reduce both beam hardening artifacts and partial volume averaging, photon-counting CT with an increased spatial and spectral resolution [20, 21], can be considered. However, due to higher costs and limited evidence on improved diagnostic accuracy compared to other CT modalities, photon-counting CT is not yet established in clinical routine [22, 23].

A negative attenuation difference was observed at high keV for the single stent, double stent and double stent + marker configuration, although for the single stent configuration the attenuation difference became less negative (− 5 HU vs. − 27.5 to − 28.5 HU) and only in a higher energy range (160–200 keV vs. 120–200 keV). These results imply a lower attenuation in the double stented lumen compared to the native vessel lumen and single stented lumen. A potential cause of the lower attenuation in the double stented lumen could be suboptimal spectral separation [11]. A dual-layer spectral CT scanner consists of two detector layers with maximal sensitivity for different energies: the inner layer mainly absorbs low-energy photons and the outer layer the remaining high-energy photons [24]. However, in practice, this setup does not provide a sharp distinction between the low- and high-energy photons, resulting in an overlap in photon energies that are absorbed by the inner and outer detector layer, especially in the mid to high photon energy range [25]. In the double stent and double stent + marker configuration, more beam hardening occurs thus removing lower energy photons that would have been detected by only the inner detector layer. This lowers the scanner’s ability to separate energies and differentiate materials, for example iodine and stent material [11]. Combined with extrapolation of the non-measured high-energy data (140–200 keV), this could result in falsely lower attenuation in the stented lumen, resulting in negative attenuation difference values. Energy separation could be improved by scanning at higher tube voltages [26], as was shown in our sub analysis comparing the attenuation difference at 120 and 140 kVp.

Our in-vitro setup presents limitations. First, PDMS was used to represent the surrounding tissue. Although the X-ray mass-attenuation coefficient of PDMS and tissue correspond well in the energy range 70–200 keV, a higher attenuation of PDMS was found for the 40–60 keV energy range (20–55%) [27, 28]. On the other hand, as the image parameters in our study follow the same trend as found in patients [12], the effect of the higher PDMS attenuation on our results is probably small. Second, the chosen set-up represented an ideal case without patient-specific anatomical differences, including vessel geometry, vessel wall, calcification and in-stent restenosis, that could affect the blooming artifact and in-stent lumen assessment as well. Moreover, in this study only one stent type and diameter was investigated, which are both known to affect blooming artifacts [5, 29]. Nevertheless, with our in-vitro setup we were able to provide a thorough analysis on the effect of VMI reconstructions on blooming artifact reduction on common stent configurations in the femoropopliteal tract. These findings will help guide clinicians in selecting the optimal reconstruction for minimizing blooming artifact and improving in-stent lumen assessment.

In conclusion, this study showed that VMI reconstructions can minimize stent-induced blooming artifacts and improve in-stent lumen assessment. For the single stent configuration, optimal scores were found in the 80–100 keV energy range, while for the double stent configuration higher energy VMI reconstructions were required. The absence of significant differences in quantitative parameters, however, suggests that the number of pixels affected by the blooming artifact is not reduced by VMI reconstructions. Comparative studies between spectral CT and photon-counting CT are indicated to evaluate their ability to reduce stent-induced blooming artifacts.

## Supplementary Information

Below is the link to the electronic supplementary material.

Supplementary file1 (DOCX 886 KB)

## Data Availability

The processed data in support of the results in this article are available within the article or supplemental materials. Unprocessed data and analysis scripts are available upon reasonable request to the corresponding author.

## Abbreviations

CFD: Computational fluid dynamics
CNR: Contrast-to-noise ratio
CT: Computed tomography
CTA: Computed tomography angiography
FWHM: Full width at half maximum
keV: Kiloelectronvolt
kVp: Kilovoltage peak
PDMS: Polydimethylsiloxane
ROI: Region of interest
SD: Standard deviation
SNR: Signal-to-noise ratio
VMI: Virtual monoenergetic imaging
