# Supplementary material for: Reducing Stent-Induced Blooming Artifacts Using Virtual Monoenergetic Imaging Reconstructions in a Superficial Femoral Artery Phantom: An In-Vitro Study
Source: Cardiovasc Eng Technol. 2026 Jul 7;17(5):591–600. doi: 10.1007/s13239-026-00848-8 (PMC13723638; doi:10.1007/s13239-026-00848-8)
Supplement: Supplementary file 1 [file 13239_2026_848_MOESM1_ESM.docx]

**Supplementary material**

Title: Reducing stent-induced blooming artifacts using virtual monoenergetic imaging reconstructions in a superficial femoral artery phantom: an in-vitro study

Journal: Cardiovascular Engineering and Technology

Authors: Lisa Rutten^a,b,c,d^, Lennart van de Velde^a,b^, Cornelis H. Slump^e^, Jasper Martens^f^, Kartik Jain^d^, Michel Versluis^c^, Michel M. P. J. Reijnen^a,b^

^a^ Multi-Modality Medical Imaging Group, TechMed Centre, University of Twente, Enschede, The Netherlands

^b^ Department of Surgery, Rijnstate, Arnhem, The Netherlands

^c^ Physics of Fluids group, TechMed Centre, University of Twente, Enschede, The Netherlands

^d^ Department of Thermal and Fluid Engineering, University of Twente, Enschede, The Netherlands

^e^ Robotics and Mechatronics group, University of Twente, Enschede, The Netherlands

^f^ Department of Radiology and Nuclear Medicine, Rijnstate, Arnhem, The Netherlands

Corresponding author: Lisa Rutten

Email address: [l.rutten@utwente.nl](mailto:l.rutten@utwente.nl)

**Fig. S1** Overview of the sampling locations for the different analysis locations: (0) native vessel, (1) single stent + marker configuration, (2) single stent configuration, (3) double stent + marker configuration, (4) double stent configuration. Five consecutive slices for the native vessel and three consecutive slices for the marker locations were selected on both ends resulting in ten and six slices for the native vessel and marker locations, respectively. For the single and double stent locations, five slices were selected at both ends with ten slices spacing to span the full analysis location. Each vertical black line corresponds to one slice and the yellow dots indicate radiopaque stent markers

**Fig. S2** Schematic overview of obtaining the full width at half maximum (FWHM). (a) A straight line was semi-automatically drawn across the diameter of the vessel. (b) From the resulting intensity profile (orange), the peaks were automatically detected (vertical lines) and the width at half the peak was determined (horizontal lines).

**Fig. S3** The attenuation difference between in-stent lumen and native vessel lumen in the single stent + marker (a) and double stent + marker (b) configurations for conventional and virtual monoenergetic image (VMI) reconstructions. The median attenuation difference is negative in the double stent + marker configuration for the 120 – 200 keV VMI image reconstructions

**Fig. S4** Median attenuation difference between in-stent lumen and native vessel in the single stent (square) and double stent (+) configuration for conventional and virtual monoenergetic image reconstructions acquired at 120 kVp (blue) and 140 kVp (red). The attenuation difference is smaller for the scans acquired at 140 kVp compared to 120 kVp. Please note that this is a different dataset

**Fig. S5** The full width at half maximum (FWHM) in the single stent + marker (a) and double stent + marker (b) configurations for conventional and virtual monoenergetic image (VMI) reconstructions. The circles are outliers defined as more than 1.5 × interquartile range away from the top or bottom of the box. * significantly larger than VMI 180 – 200 keV (p < 0.05)

**Table S1** Mean image quality scores on stent-lumen interface (Likert scale 1 – 5) based on three independent readers for conventional and virtual monoenergetic image (VMI) reconstructions

|  | iDose | 40 keV | 60 keV | 70 keV | 80 keV | 90 keV | 100 keV | 140 keV |
| --- | --- | --- | --- | --- | --- | --- | --- | --- |
| Single | 3.00 | 1.00 | 2.00 | 2.00 | 3.67 | 4.00 | 4.00 | 3.33 |
| Single + marker | 2.67 | 1.00 | 2.33 | 2.67 | 3.33 | 4.00 | 3.67 | 3.67 |
| Double | 2.33 | 1.00 | 2.00 | 2.00 | 3.33 | 2.00 | 3.67 | 4.00^*^ |
| Double + marker | 2.33 | 1.00 | 2.00 | 2.00 | 3.00 | 3.33 | 3.33 | 3.00 |

The highest mean subjective score per stent configuration is underlined. * significantly higher than VMI 40 keV.

**Table S2** Mean image quality scores on blooming artifact (Likert scale 1 – 5) based on three independent readers for conventional and virtual monoenergetic image reconstructions

|  | iDose | 40 keV | 60 keV | 70 keV | 80 keV | 90 keV | 100 keV | 140 keV |
| --- | --- | --- | --- | --- | --- | --- | --- | --- |
| Single | 2.33 | 1.00 | 1.67 | 2.67 | 3.67 | 3.67 | 4.00 | 3.67 |
| Single + marker | 2.00 | 1.00 | 2.00 | 2.67 | 3.33 | 4.00 | 3.67 | 4.00 |
| Double | 1.67 | 1.00 | 1.33 | 2.00 | 3.00 | 3.00 | 4.00 | 4.33 |
| Double + marker | 1.67 | 1.00 | 1.33 | 2.00 | 3.00 | 3.67 | 3.33 | 4.00 |

The highest mean subjective score per stent configuration is underlined.

**Table S3** Mean image quality scores on overall image quality (Likert scale 1 – 5) based on three independent readers for conventional and virtual monoenergetic image reconstructions

|  | iDose | 40 keV | 60 keV | 70 keV | 80 keV | 90 keV | 100 keV | 140 keV |
| --- | --- | --- | --- | --- | --- | --- | --- | --- |
| Single | 3.00 | 1.00 | 2.33 | 2.33 | 3.67 | 3.67 | 4.00 | 3.00 |
| Single + marker | 2.33 | 1.00 | 2.00 | 3.00 | 3.33 | 4.00 | 3.67 | 3.67 |
| Double | 2.00 | 1.00 | 2.00 | 2.33 | 3.00 | 2.67 | 3.33 | 3.67 |
| Double + marker | 2.00 | 1.00 | 2.00 | 2.33 | 3.00 | 3.33 | 3.00 | 3.00 |

The highest mean subjective score per stent configuration is underlined.
